# Ontario Veterinary Association

**Published:** 1895-01

**Authors:** C. M. Sweetapple

**Affiliations:** Secretary


Secretary.

### ONTARIO VETERINARY COLLEGE, TORONTO, CANADA.

### THE ONTARIO VETERINARY ASSOCIATION.

THE Annual Meeting of the Ontario Veterinary Association was held in the Veterinary College, Toronto, Canada, on Friday, December 21, 1894.

The President, Mr. W. Burns, V.S., opened the meeting with a few well-chosen remarks, and called for the reading of the reports.

The Secretary's, Registrar's, and Auditors' reports were then received and adopted.

Considerable discussion then ensued on the action of certain parties in issuing so-called veterinary dental diplomas. The issuing of these was very strongly condemned at the last meeting, tending, as it does, to bring legitimate veterinary science into disrepute, reputable members of the veterinary profession considering this so-called veterinary dentistry as a complete humbug. Mr. John Wende, V.S., also a member of the New York State Veterinary Association, remarked that that association also very strongly condemned the issuing of these veterinary dental diplomas.

Major Lloyd, Mr. Gibbs, Mr. C. Elliott, Mr. O'Neil, and others took part in this discussion, and it was ultimately resolved "That the same committee that were appointed last year should be continued, and that their efforts should be directed to suppress this humbug.

Mr. W. J. Wilson, V.S., of London, read an excellent paper on "The Dangers of Using the Meat and also the Milk of Diseased Animals as Human Food." He called attention to the toxic properties produced by the germ of tuberculosis (the bacillus tuberculosis) being especially injurious to consumptive patients, and that these products were still existing in the meat and milk, although the vitality of the bacillus itself may have been destroyed by cooking or otherwise. He recommended the establishment of public abattoirs and the inspection of meat by qualified men. He also recommended that dairies should be placed under suitable inspection, and that the hygienic conditions of milch cows should be looked to, cleanliness and sufficient air space being essential.

In the discussion that followed, in which Major Lloyd, Messrs. Shaw, Cowan, and others took part, it was remarked that the "tuberculin test" was a reliable diagnostic agent, but judgment must be used in applying it; that it is well to take the body temperature of other animals in the herd, not the injected, as variations in the temperature may be produced by accidental causes.

Mr. Cowan, Veterinary Inspector, said that it was well not to make unnecessary alarm in connection with tuberculosis; that the disease existed only to a slight extent in Canada amongst cattle—less than in most other countries—and that the disease was on the decrease here. He also said that the various Boards of Health have great power in looking into the milk supply; that there is all the law that is required in investigating the milk and meat supply, and in condemning tubercular cases.

The thanks of the meeting were voted to Mr. W. J. Wilson for his interesting paper.

Mr. A. Crowforth, V.S., of Lockport, N. Y., read an interesting and exhaustive paper on "Tuberculosis in Relation to Animal Industry and Public Health; its Prevalence and Importance." He said that it prevailed so extensively throughout the civilized world that no disease is so deserving of close study or of the enforcement of effective measures for its suppression. Cholera, yellow fever, and smallpox, which occasionally appear, creating universal terror and dismay, claim but few victims in comparison with this ever-present and universally devastating malady. These other plagues are quick, severe, and fatal, and therefore can be promptly recognized and even stamped out; whereas tuberculosis is slow and uncertain in its progress, and often escapes recognition for a long time. It may be classed with "*the pestilence that walketh in darkness*," while the other diseases named may be likened to the "*destructions that waste at noonday*." He gave statistics showing the deaths in various parts of the world in the human race, and mentioned its prevalence as the same disease in the domestic animals, produced by the same micro-organism (the bacillus tuberculosis), and the difficulty of obtaining reliable statistics.

In the Middle Ages tuberculosis in animals was recognized as contagious, and laws were made against the use of affected carcasses as human food, which remain in force in Spain and Italy to the present day. In the early part of the present century its contagious character was doubted by medical practitioners; but at the present time, in the light of numerous investigations and experiments, all candid scientific observers accept the doctrine of its contagious character. He described the germ, its history, and mode of propagation, and also the accessory causes which tend to produce the disease. But some of these causes can produce the disease in the absence of the *bacillus*. He described the disease in the various organs and tissues of the body, and mentioned the difficulties experienced in diagnosis. He spoke strongly in favor of "tuberculin" as a diagnostic agent, and said those who had used it most valued it the most highly. In conclusion, he compared the geographic distribution of cattle and the prevalence of tuberculosis in the human race, and showed the ultimate relations of cattle to man as a potent agent in the extension and maintenance of consumption in the human family.

A discussion followed, in which Messrs. O'Neil, Wilson, and others took part, and a vote of thanks was tendered to Mr. Crowforth for his interesting and exhaustive paper.

Prof. Smith exhibited from the Museum of the Veterinary College specimens of "gangrenous ergotism" of the legs of cattle from cases he had investigated last spring.

The sum of $25 was appropriated for a medal to be competed for by students of the Ontario Veterinary College at the approaching spring examinations.

The following are the officers for the ensuing year:

*President.*—Mr. G. L. Robson.

*Vice-Presidents.*—H. Hopkins and D. Hamilton.

*Secretary.*—C. M. Sweetapple.

*Treasurer.*—W. Cowan.

*Auditors.*—Messrs. J. D. O'Neil and C. Elliott.

*Directors.*—Messrs. J. Wende, W. Burns, J. F. Quin, W. Gibb, W. J. Wilson, S. Holder, A. Crowforth, and W. Steel.

C. M. SWEETAPPLE,
Secretary.

